# Patent foramen ovale closure versus medical therapy for prevention of recurrent ischemic events in patients with cryptogenic stroke or transient ischemic attack and documented patent foramen ovale: a systematic review and network meta-analysis of randomized controlled trials and observational studies

**DOI:** 10.3389/fcvm.2026.1785684

**Published:** 2026-08-12

**Authors:** Yuanpeng Deng, Di An, Xiaoman Xiong, Zhengmei Zhou, Xingde Liu, Yi Xiang

**Affiliations:** 1 The Second Clinical Medical College, Guizhou University of Traditional Chinese Medicine, Guiyang, Guizhou, China; 2 The Key Laboratory of Myocardial Remodeling Research, The Affiliated Hospital of Guizhou Medical University, Guiyang, Guizhou, China; 3 Department of Cardiovascular Internal Medicine, Second Hospital, Guizhou University of Traditional Chinese Medicine, Guiyang, Guizhou, China

**Keywords:** bleeding, cryptogenic stroke, network meta-analysis, patent foramen ovale, recurrent stroke, risk ratio

## Abstract

**Background:**

This network meta-analysis (NMA) aimed to compare the efficacy and safety of patent foramen ovale (PFO) closure and medical therapy categories, including antiplatelet therapy, anticoagulation, and mixed medical therapy.

**Methods:**

We systematically searched PubMed, Embase, the Cochrane Library, and Web of Science from inception to June 22, 2026. Eligible randomized controlled trials and observational studies comparing PFO closure with medical therapy in adult patients with cryptogenic ischemic stroke or transient ischemic attack and confirmed PFO were included.

**Results:**

Eighteen studies involving 7,135 participants, including six randomized controlled trials and 12 observational studies, were included. Four intervention nodes were analyzed: PFO closure, antiplatelet therapy, anticoagulation, and mixed medical therapy. For recurrent cerebrovascular ischemic events, PFO closure was associated with a lower risk than antiplatelet therapy (RR 0.22, 95% CI 0.12–0.41), anticoagulation (RR 0.40, 95% CI 0.19–0.82), and mixed medical therapy (RR 0.51, 95% CI 0.33–0.77). For bleeding events, PFO closure was associated with a significantly lower risk than anticoagulation (RR 0.16, 95% CI 0.06-0.42), while the comparisons with antiplatelet therapy (RR 0.49, 95% CI 0.20-1.23) and mixed medical therapy (RR 0.91, 95% CI 0.46-1.79) did not reach statistical significance. Model-fit assessment showed similar DIC values between the consistency and unrelated mean-effects models.

**Conclusions:**

PFO closure showed the most favorable efficacy profile for preventing recurrent cerebrovascular ischemic events and was associated with a significantly lower bleeding risk than anticoagulation. However, safety findings for comparisons with antiplatelet therapy and mixed medical therapy should be interpreted cautiously because bleeding events were sparse and confidence intervals were wide.

**Systematic Review Registration:**

CRD420251148908

## 1 Introduction

Ischemic stroke and transient ischemic attack (TIA) remain major causes of disability and death worldwide (1). A substantial proportion of cases are classified as cryptogenic stroke, meaning that no definite thrombotic source is identified after standard etiologic evaluation (2). Patent foramen ovale (PFO), one of the most common congenital interatrial communications, is prevalent in the general population; when a right-to-left shunt (RLS) is present, venous thrombi can traverse the interatrial septum into the arterial circulation and trigger paradoxical embolism (3). Prior observations indicate that certain anatomical characteristics—such as atrial septal aneurysm, large shunt, and tunnel-type PFO—are associated with a higher risk of recurrent ischemic events (4). Consequently, determining effective secondary prevention strategies for patients with cryptogenic stroke/TIA and concomitant PFO has become a key issue in clinical practice (5).

Current secondary prevention for patients with cryptogenic stroke/TIA and documented PFO mainly includes two approaches: percutaneous PFO closure, which anatomically eliminates the right-to-left shunt, and medical therapy, including antiplatelet therapy or anticoagulation (6). However, available studies differ in patient selection, PFO anatomical risk features, comparator regimens, outcome definitions, and follow-up duration (7–9). Definitions and grading of bleeding are also inconsistent across studies, although ISTH-based criteria are commonly referenced in antithrombotic trials (10–12). In addition, differences in PFO imaging confirmation methods, including TEE, contrast-TTE, and TCD, may further contribute to heterogeneity (13). Together with accumulating evidence from different regions and populations, these variations make it difficult to directly compare treatment strategies and to balance recurrence prevention against bleeding risk in clinical decision-making (14).

Against this background, traditional systematic reviews and pairwise meta-analyses can address specific head-to-head questions but are limited in providing cross-strategy integration and unified ranking within a single methodological framework. Clinical decision-making often requires balancing efficacy (reducing recurrence) against safety (bleeding burden), yet existing syntheses rarely present relative risks across multiple strategies using a harmonized safety endpoint. To address these gaps, this study aims to: (1) conduct a network meta-analysis within a unified framework, incorporating randomized controlled trials and observational cohorts to integrate direct and indirect evidence comparing PFO closure, antiplatelet therapy, anticoagulation, and mixed medical therapy; (2) use the risk ratio (RR) as a common effect measure, focusing on two key outcomes—recurrent cerebrovascular ischemic events (primary efficacy endpoint) and any bleeding (safety endpoint)—to enhance comparability across studies; (3) provide treatment rankings under a random-effects consistency model and examine robustness via risk-of-bias assessment and consistency/heterogeneity diagnostics; and (4) incorporate the latest follow-up data and Asian cohort evidence where possible to improve generalizability and translational relevance. Our goal was to provide a clearer benefit–risk framework for PFO closure versus medical therapy in this population.

## 2 Methods

### 2.1 Inclusion and exclusion criteria

#### 2.1.1 Inclusion criteria

- **Study design**: Randomized controlled trials (RCTs) or observational studies (prospective or retrospective cohort studies) with a clear study design that reported comparative outcomes between PFO closure and medical therapy.
- **Participants**: Adult patients (aged ≥18 years) with cryptogenic ischemic stroke or transient ischemic attack (TIA), with PFO confirmed by transesophageal echocardiography (TEE) or other standard imaging modalities.
- **Interventions**: Closure group: Patients who underwent percutaneous or surgical PFO closure; Medical therapy group: Patients treated with antiplatelet or anticoagulant therapy. Eligible studies were required to include a direct comparison between PFO closure and medical therapy in patients with documented PFO.
- **Outcomes**: Primary efficacy outcome: Recurrent cerebrovascular ischemic events, including ischemic stroke, TIA, or composite cerebrovascular outcomes as reported by the study. If only “stroke recurrence” or “composite ischemic events” were available, the most comprehensive endpoint with the highest number of events was included. Safety outcomes: Bleeding events, including major or minor bleeding; when studies did not distinguish severity, all reported bleeding events were included.
- **Language and publication date**: Studies published in full-text English, with no restriction on publication date.

#### 2.1.2 Exclusion criteria

- **Non-relevant study types**: Case reports, case series, expert opinions, editorials, reviews, or purely anatomical/mechanistic imaging studies without clinical outcome data. Studies comparing antiplatelet therapy with anticoagulation alone without a PFO closure group were excluded.
- **Ineligible population**: Patients without cryptogenic stroke; age <18 years; patients with other defined stroke etiologies (e.g., atherosclerosis, atrial fibrillation) or with complex cardiac anomalies such as atrial septal defects.
- **Unavailable data**: Studies without a comparison group, without reportable clinical outcomes, or from duplicate publications without additional data.

### 2.2 Literature search

We conducted a systematic search of PubMed, Embase, the Cochrane Library, and Web of Science from inception to June 22, 2026. Search terms included “Foramen Ovale, Patent,” “Stroke,” and “Ischemic Attack, Transient,” combined with related synonyms and controlled vocabulary terms such as MeSH and Emtree where applicable. The detailed search strategies for each database are provided in Supplementary Material 1.

### 2.3 Literature selection and data extraction

All search results were imported into EndNote X9 for de-duplication. Two reviewers then independently screened titles and abstracts to exclude studies that clearly did not meet the inclusion criteria; potentially eligible records were retrieved in full and reassessed against the prespecified inclusion and exclusion criteria. Disagreements were resolved by discussion or adjudication by a third reviewer. For studies ultimately included, two reviewers independently extracted data using a standardized form, covering: (i) study characteristics—first author, year of publication, country, study design, and data source; (ii) population characteristics—sample size, age, and interventions; (iii) primary outcome—recurrent cerebrovascular ischemic events (stroke, TIA, or the composite endpoint); (iv) safety outcomes—major or minor bleeding (if not distinguished in the original report, treated as “any bleeding”); and (v) follow-up duration and the number of outcome events. Extracted data were cross-checked and entered into the statistical analysis database; where key information was missing, we attempted to contact the original authors or derived the data through indirect calculations. When available, information on specific antithrombotic regimens was extracted. However, because many studies reported outcome events at the treatment-class level rather than by individual drug, the network meta-analysis was conducted using intervention categories, including antiplatelet therapy, anticoagulation, and mixed medical therapy.

### 2.4 Quality assessment of included studies

Risk of bias and study quality were assessed as follows. For randomized controlled trials (RCTs), we applied the Cochrane Risk of Bias 2.0 (RoB 2) tool (15) across five domains—randomization process, deviations from intended interventions, missing outcome data, measurement of the outcome, and selection of the reported result. Each RCT was judged at the domain and overall level as low risk, some concerns, or high risk. For observational studies, we used the Newcastle–Ottawa Scale (NOS) (16), scoring up to 9 points across three categories: selection (0–4), comparability (0–2), and outcome assessment (0–3). Studies scoring ≥7 were classified as high quality, 5–6 as moderate quality, and <5 as low quality. All assessments were performed independently by two reviewers and cross-checked; disagreements were resolved by discussion or adjudication by a third reviewer.

### 2.5 Statistical analysis

Network meta-analyses and related figures were generated in R (version 4.1.3) using the gemtc package (version 4.1.3), which implements Bayesian network meta-analysis models based on Markov chain Monte Carlo methods (17). All included outcomes were binary; effect sizes were expressed as risk ratios (RRs) with 95% confidence intervals (CIs). For each outcome, we fitted a random-effects consistency model and evaluated model fit and the plausibility of the consistency assumption using the Deviance Information Criterion (DIC); lower DIC indicates better fit, and a DIC difference <5 between the consistency and inconsistency models was interpreted as evidence of good consistency (18, 19). A Bayesian Markov chain Monte Carlo (MCMC) framework was used to estimate the network models, running two chains with 10,000 burn-in iterations followed by 50,000 sampling iterations. Network plots were produced separately for efficacy and safety outcomes. To rank interventions, we calculated treatment ranking probabilities, where higher values indicate better performance for efficacy or safety, respectively (20). All statistical tests were two-sided, with *P* < 0.05 considered statistically significant.

## 3 Results

### 3.1 Literature screening results

**Figure 1:**
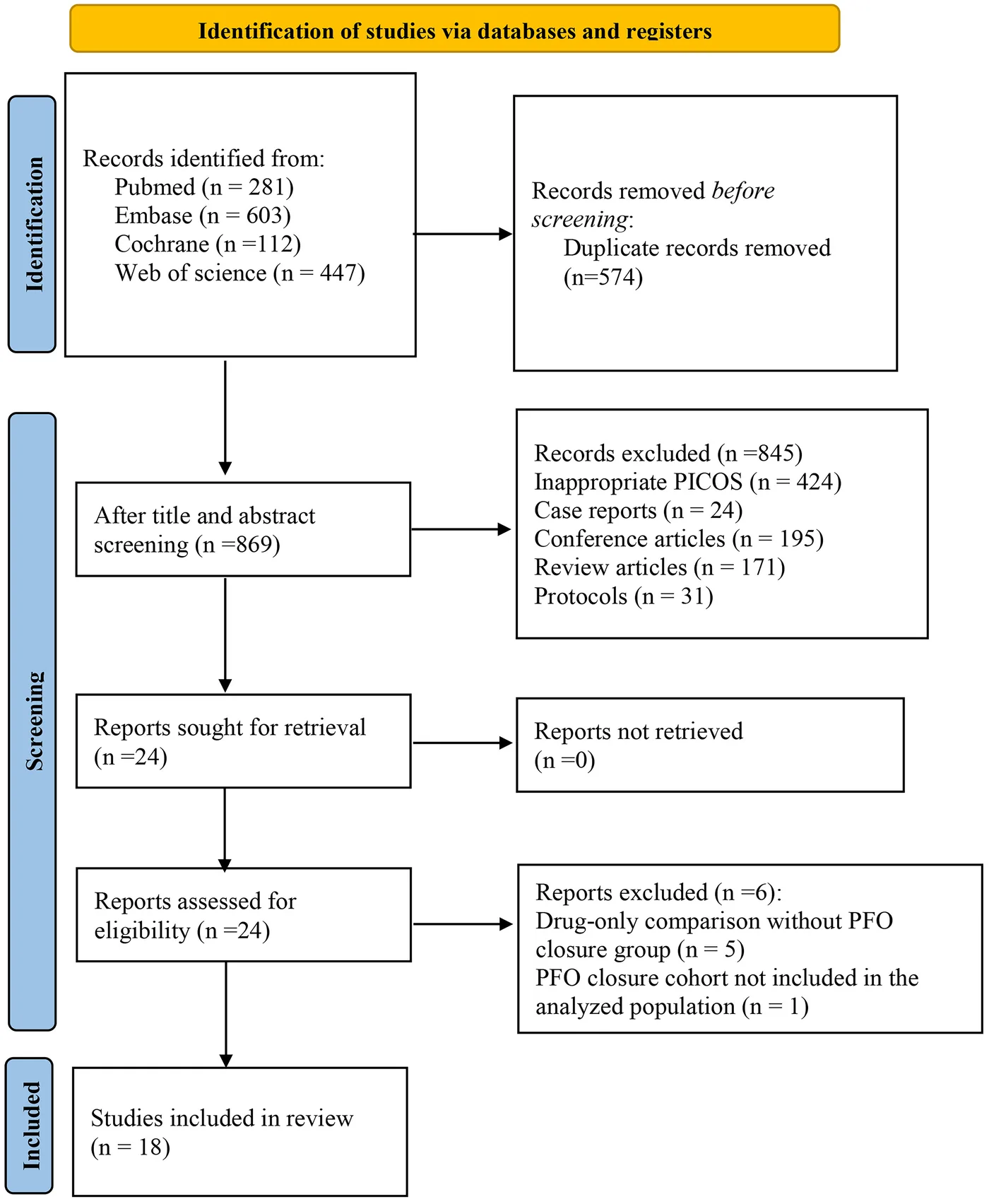
Literature screening flowchart. PICOS indicates Population, Intervention, Comparator, Outcomes, and Study design. Records excluded for inappropriate PICOS were those that did not meet the predefined eligibility criteria regarding target population, intervention/comparator, clinical outcomes, or eligible study design. Full-text exclusion reasons are shown in the flow diagram.

The database search identified 1,443 records, including 281 from PubMed, 603 from Embase, 112 from the Cochrane Library, and 447 from Web of Science. After removing 574 records before screening, 869 records remained for title and abstract screening. Twenty-four reports were assessed for eligibility, of which six were excluded after full-text review. Finally, 18 studies were included in the meta-analysis, including six randomized controlled trials and 12 observational studies (Figure 1).

### 3.2 Characteristics of included studies

This meta-analysis included 18 studies (8, 9, 14, 21–35) published between 2004 and 2024. Most were multicenter investigations from Europe/North America and Asia (e.g., France, Germany, the United States, Canada, Korea, and China), supplemented by several single-center cohort studies. All participants were patients with cryptogenic ischemic stroke or transient ischemic attack (TIA) and imaging-confirmed patent foramen ovale (PFO); several studies specifically enrolled high-risk PFO populations (large shunt or atrial septal aneurysm). Interventions comprised comparisons of PFO closure (percutaneous or surgical) versus medical therapy. In studies with treatment-specific medical arms, antiplatelet therapy and anticoagulation were extracted separately; when the original study reported only an overall medical-therapy arm without extractable treatment-specific event counts, the arm was classified as mixed medical therapy. Sample sizes varied widely—from small single-center cohorts (approximately 100 patients) to multicenter RCTs (nearly 5,400 participants)—with follow-up durations ranging from 14 months to more than 5 years. Detailed baseline characteristics and study information are provided in Table 1. To improve transparency and allow readers to verify the event burden contributing to the network meta-analysis, we provided the raw event data for recurrent ischemic and bleeding outcomes by study and treatment arm in Supplementary Material 2.

**Table 1:** Baseline characteristics of the included studies.

Study	Year	Country	study type	Sample source	treatment	Sample size	Age	Follow up	Disease	Outcomes
Mas JL et al. (8)	2017	France and Germany	RCT	multicenter	PFO_closure vs Antiplatelet vs Anticoagulant	238/235/187	43.9 ± 10.7	Mean 5.3 ± 2.0 years	Cryptogenic stroke, large shunt/atrial septal aneurysm	①②
Lee PH et al. (18)	2018	South Korea	RCT	multicenter	PFO_closure vs Mixed medical therapy	60/60	Mean 51.8	Median 2.8 years	Cryptogenic stroke with high-risk PFO	①②
Furlan AJ et al. (9)	2012	United States and Canada	RCT	multicenter	PFO_closure vs Mixed medical therapy	447/462	≈46	2 years	Cryptogenic stroke/TIA with PFO	①②
Saver JL et al. (21)	2017	United States and Canada	RCT	multicenter	PFO_closure vs Mixed medical therapy	499/481	45.9 ± 9.9	Median 5.9 years	Cryptogenic ischemic stroke with PFO	①②
Søndergaard L et al. (30)	2017	Multinational	RCT	multicenter	PFO_closure vs Antiplatelet	441/223	Mean 45.2	Median 3.2 years	Cryptogenic stroke with PFO	①②
Meier B et al. (31)	2013	Multinational	RCT	multicenter	PFO_closure vs Mixed medical therapy	204/210	44.3/44.6	Mean 4.1/4.0 years	Cryptogenic embolism with PFO	①②
Casaubon L et al. (22)	2007	Canada	Cohort	multicenter	PFO_closure vs Antiplatelet vs Anticoagulant	60/41/20	Median 43	Median 32 months	Cryptogenic stroke/TIA with PFO	①
Paciaroni M et al. (23)	2011	Italy	Cohort	multicenter	PFO_closure vs Antiplatelet vs Anticoagulant	121/93/24	42.2 ± 10.0	2 years	Cryptogenic minor stroke/TIA with PFO	①
Schuchlenz HW et al. (24)	2005	Austria	Cohort	single-center	PFO_closure vs Antiplatelet vs Anticoagulant	167/66/47	NR	Mean 2.6 years	Cryptogenic cerebrovascular events with PFO	①②
Thanopoulos BV et al. (25)	2006	Greece	Cohort	single-center	PFO_closure vs Antiplatelet	48/44	43 ± 11/40 ± 12	24 months	Cryptogenic stroke with PFO	①②
Windecker S et al. (26)	2004	Switzerland	Cohort	single-center	PFO_closure vs Antiplatelet vs Anticoagulant	150/79/79	≈50	Mean 2.1/2.4 years	Cryptogenic stroke/TIA with PFO	①
Low CE et al. (27)	2023	Singapore	Cohort	single-center	PFO_closure vs Mixed medical therapy	84/148	44.3 ± 10.8	Median 1486 days	Cryptogenic ischemic stroke with PFO	①
Lee PH et al. (28)	2024	South Korea	Cohort	multicenter	PFO_closure vs Mixed medical therapy	161/276	Mean 68.1	Median 3.9 years	Cryptogenic stroke in patients aged ≥60 with high-risk PFO	①②
Chen PL et al. (29)	2023	China	Cohort	single-center	PFO_closure vs Mixed medical therapy	97/76	56.5 ± 14.9	Mean 2.5 years	Cryptogenic stroke/TIA with PFO	①
Pezzini A et al. (32)	2016	Italy	Cohort	multicenter	PFO_closure vs Mixed medical therapy	206/315	35.3 ± 7.4/35.7 ± 6.8	Median 32.0/36.0 months	First-ever cryptogenic ischemic stroke with PFO	①
Danese A et al. (33)	2017	Italy	Cohort	single-center	PFO_closure vs Mixed medical therapy	77/82	49.7 ± 9.7	Mean 51.6 ± 34.8 months	Cryptogenic stroke with PFO	①
Liu K et al. (34)	2020	United States/China	Cohort	multicenter	PFO_closure vs Mixed medical therapy	383/208	≈50	Median 53 months	PFO-attributable cryptogenic embolism	①
Poli S et al. (35)	2021	Germany	Cohort	single-center	PFO_closure vs Mixed medical therapy	146/90	Median 58	Mean 2.8 ± 1.3 years	Cryptogenic ischemic stroke/TIA with PFO	①②

①: Recurrent cerebrovascular ischemic events (including stroke or TIA); ②: Any bleeding event.

### 3.3 Risk of bias

**Figure 2:**
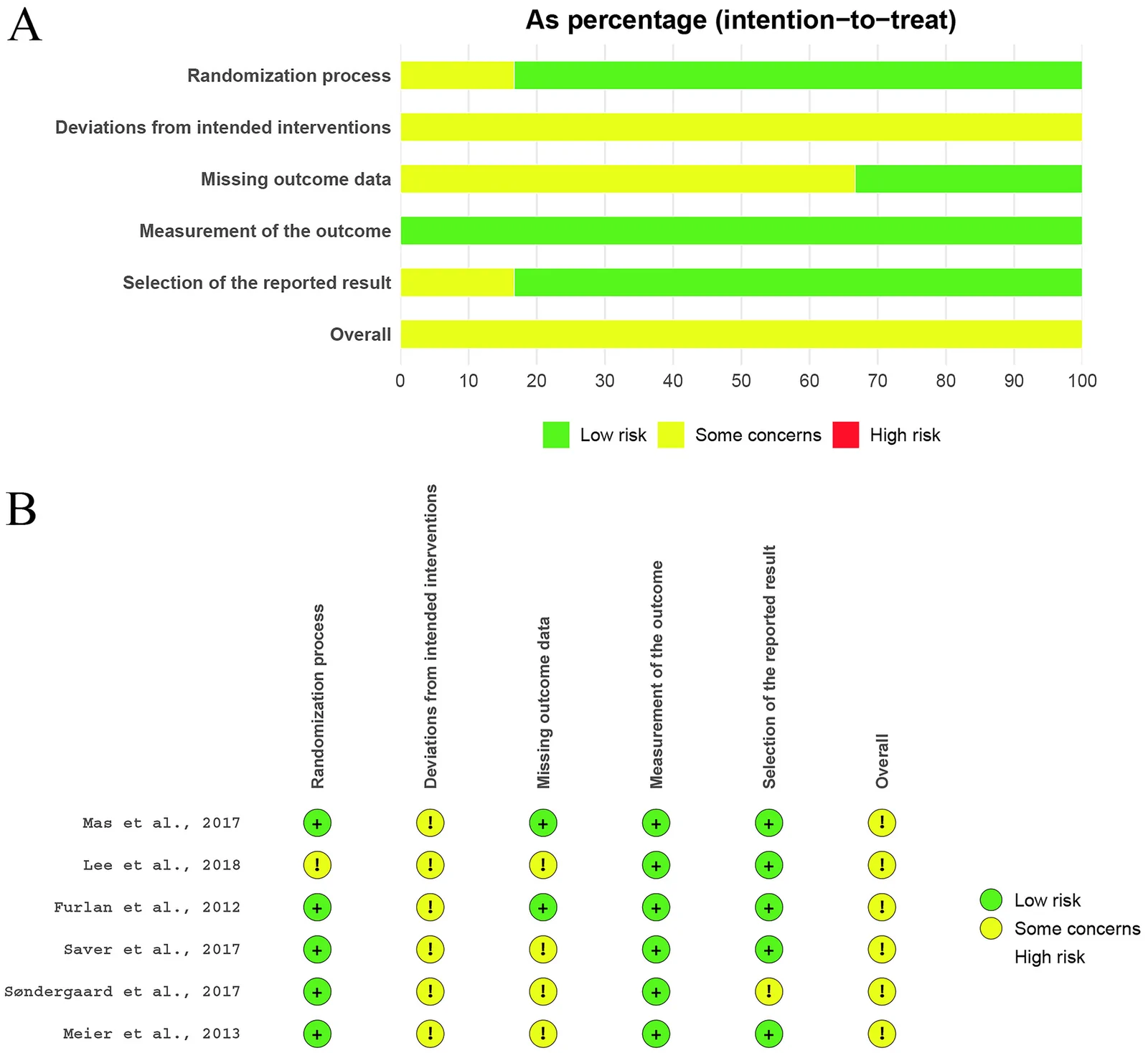
Assessment of the quality of randomized controlled trials. **(A)** Risk of bias summary; **(B)** Risk of bias graph.

All six randomized controlled trials (RCTs) were evaluated using the Cochrane Risk of Bias 2 (RoB 2) tool. Most trials showed low risk for outcome measurement, while deviations from intended interventions and overall judgments were mainly rated as some concerns (Figure 2). The 12 observational studies were appraised with the Newcastle–Ottawa Scale (NOS; maximum 9 points). All scored ≥7 (high quality), with several recent studies applying propensity-score matching or multivariable adjustment to mitigate selection bias. Overall, the quality of the observational evidence was acceptable, supporting the robustness of our findings (Table 2).

**Table 2:** Newcastle–Ottawa scale quality assessment of observational studies.

Study	Exposed cohort	Non-exposed cohort	Exposure	No baseline outcome	Comparability	Outcome assessment	Follow-up length	Follow-up adequacy	Total
Paciaroni M et al., 2011 (23)	1	1	1	1	1	1	1	1	8
Schuchlenz HW et al., 2005 (24)	1	1	1	1	1	1	1	1	8
Thanopoulos BV et al., 2006 (25)	1	1	1	1	1	1	1	0	7
Windecker S et al., 2004 (26)	1	1	1	1	2	1	1	1	9
Low CE et al., 2023 (27)	1	1	1	1	2	1	1	1	9
Lee PH et al., 2024 (28)	1	1	1	1	2	1	1	1	9
Chen PL et al., 2023 (29)	1	1	1	1	2	1	1	1	9
Casaubon L et al., 2007 (22)	1	1	1	1	1	1	1	0	7
Pezzini A et al., 2016 (32)	1	1	1	1	2	1	1	1	9
Danese A et al., 2017 (33)	1	1	1	1	1	1	1	0	7
Liu K et al., 2020 (34)	1	1	1	1	2	1	1	1	9
Poli S et al., 2021 (35)	1	1	1	1	1	1	1	1	8

### 3.4 Network meta-analysis results

#### 3.4.1 Network structure and model fit

**Figure 3:**
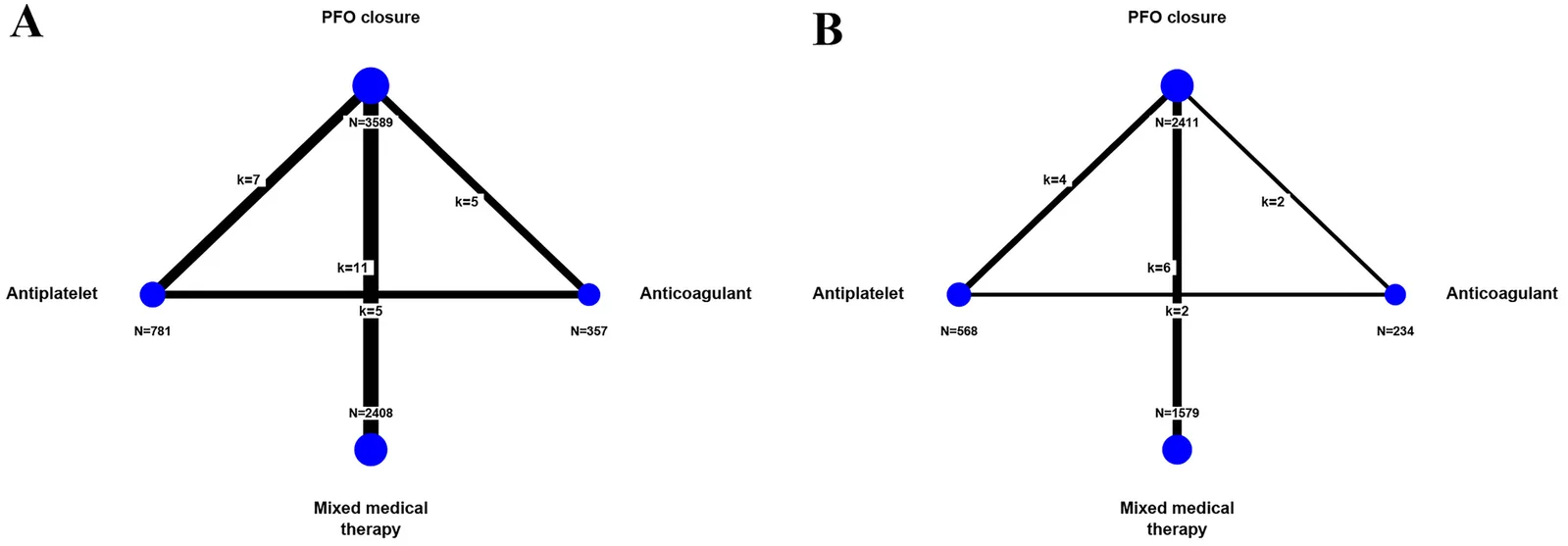
Network diagrams for the efficacy and safety outcomes. **(A)** Network diagram for recurrent cerebrovascular ischemic events. Nodes represent intervention categories, and node size is proportional to the number of participants contributing to each intervention node. Solid lines represent direct comparisons between interventions, and line thickness is proportional to the number of direct comparison studies. **(B)** Network diagram for bleeding events. Nodes and lines are interpreted in the same way as in panel A. Indirect comparisons were estimated through the network meta-analysis model and are reported in the league tables rather than being drawn as additional network edges, to avoid implying direct head-to-head evidence where none existed.

The network comprised four intervention nodes: PFO closure, antiplatelet therapy, anticoagulation, and mixed medical therapy. Mixed medical therapy was used only when the original study reported a medical-treatment arm without extractable event counts for specific antithrombotic regimens. Standalone studies comparing antiplatelet therapy with anticoagulation without a PFO closure arm were not included in the network. These nodes formed connected networks for both efficacy and safety outcomes (Figure 3A–B). The DIC difference between the random-effects consistency model and the inconsistency model was <5, indicating good model fit and supporting the validity of the consistency assumption (Supplementary Material 3).

#### 3.4.2 Primary efficacy outcome: recurrent cerebrovascular ischemic events

**Figure 4:**
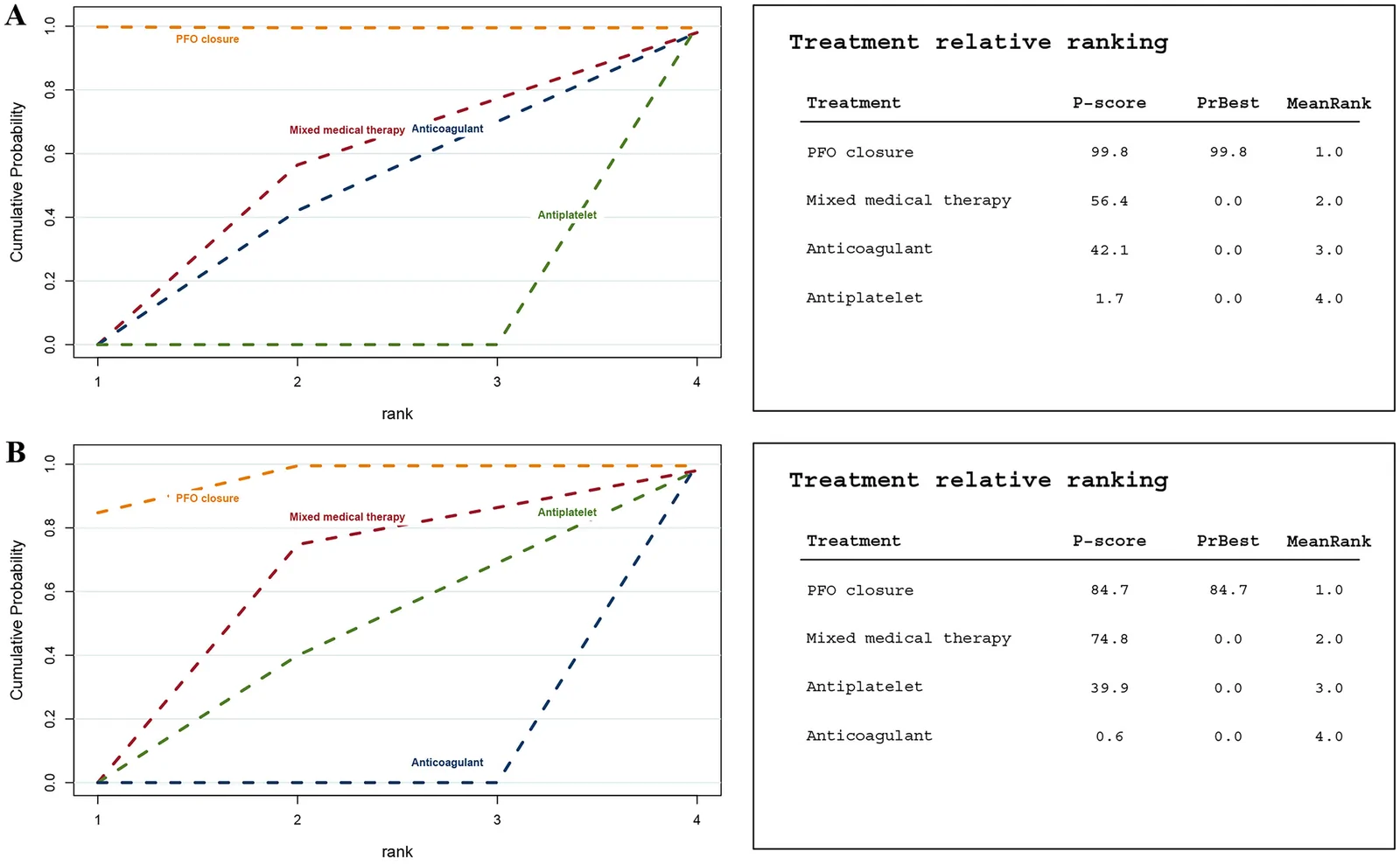
Treatment ranking curves and P-score ranking. **(A)** Cumulative ranking curve of efficacy outcomes (recurrence of ischemic events). **(B)** Cumulative ranking curve for safety outcomes (bleeding events).

The network meta-analysis included 18 studies (8, 9, 14, 21–35). Compared with antiplatelet therapy, PFO closure was associated with a lower risk of recurrent cerebrovascular ischemic events (RR 0.22, 95% CI 0.12–0.41). PFO closure was also associated with a lower risk compared with anticoagulation (RR 0.40, 95% CI 0.19–0.82) and mixed medical therapy (RR 0.51, 95% CI 0.33–0.77). Antiplatelet therapy showed a higher, non-significant direction of recurrent-event risk than anticoagulation (RR 1.78, 95% CI 0.95–3.34) and a higher direction than mixed medical therapy (RR 2.26, 95% CI 1.07–4.73) (Table 3). Overall ranking indicated that PFO closure had the highest probability of being best for preventing recurrence, followed by mixed medical therapy, anticoagulation, and antiplatelet therapy (Figure 4A). Medical-therapy-node comparisons are presented for completeness and should be interpreted as secondary indirect estimates; the primary focus of this analysis remains PFO closure versus medical therapy.

**Table 3:** League table for the rate of recurrent cerebrovascular ischemic events; RR (95% CI). Each cell represents the column-defining treatment versus the row-defining treatment.

PFO_closure	Antiplatelet	Anticoagulant	Mixed medical therapy
**0.22** **(0.12, 0.41)**			
**0.40** (**0.19, 0.82)**	1.78 (0.95, 3.34)		
**0.51** (**0.33, 0.77)**	**2.26 (1.07, 4.73)**	1.26 (0.55, 2.91)	

Bold values indicate estimates with 95% confidence intervals not crossing 1.00.

#### 3.4.3 Safety outcome: bleeding risk

The network meta-analysis for bleeding events included studies with extractable bleeding data (8, 9, 14, 21, 24, 25, 28, 30, 31, 35). Compared with antiplatelet therapy, PFO closure showed a lower direction of bleeding risk (RR 0.49, 95% CI 0.20–1.23). PFO closure was associated with a lower bleeding risk than anticoagulation (RR 0.16, 95% CI 0.06–0.42) and had a similar direction compared with mixed medical therapy (RR 0.91, 95% CI 0.46–1.79). Antiplatelet therapy showed a lower bleeding risk than anticoagulation (RR 0.32, 95% CI 0.11–0.89), whereas mixed medical therapy had a higher direction of bleeding risk than antiplatelet therapy (RR 1.84, 95% CI 0.59–5.73) (Table 4). Overall ranking indicated the lowest bleeding risk with PFO closure, followed by mixed medical therapy, antiplatelet therapy, and anticoagulation (Figure 4B). Medical-therapy-node comparisons are presented for completeness and should be interpreted cautiously, because the primary focus of this analysis remains PFO closure versus medical therapy.

**Table 4:** League table for the rate of safety endpoints; RR (95% CI). Each cell represents the column-defining treatment versus the row-defining treatment.

PFO_closure	Antiplatelet	Anticoagulant	Mixed medical therapy
0.49 (0.20, 1.23)			
**0.16** **(0.06, 0.42)**	**0.32 (0.11, 0.89)**		
0.91 (0.46, 1.79)	1.84 (0.59, 5.73)	**5.74 (1.76, 18.71)**	

Bold values indicate estimates with 95% confidence intervals not crossing 1.00.

### 3.5 Publication bias

**Figure 5:**
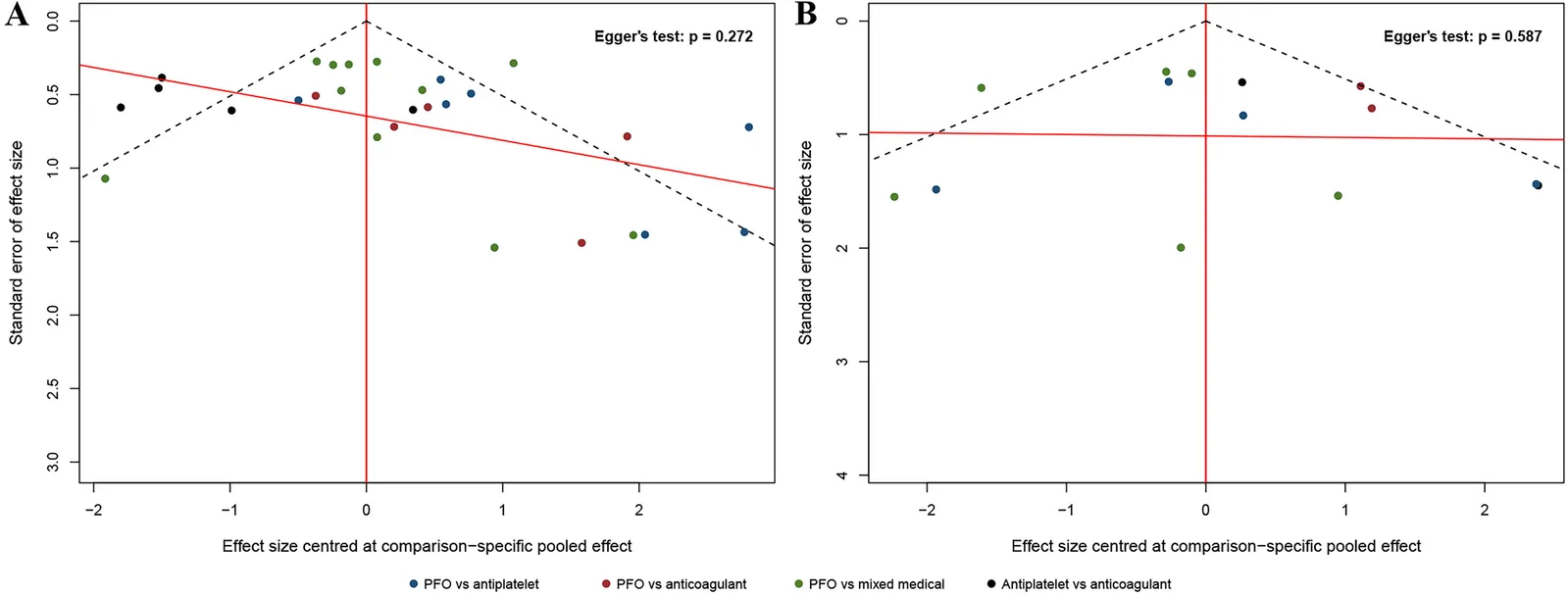
Comparison-adjusted funnel plots for assessment of small-study effects and publication bias. **(A)** Recurrent cerebrovascular ischemic events. **(B)** Any bleeding events. Points represent study-level comparisons, colors indicate comparison types, and Egger's test *p* values are shown in each panel.

Visual inspection of the funnel plots showed approximate symmetry, indicating no substantial publication bias or small-study effects. Overall, the findings appear robust, with no appreciable systematic bias (Figure 5).

## 4 Discussion

This study incorporated six randomized controlled trials and 12 observational studies, and used a network meta-analysis to evaluate PFO closure versus medical therapy in patients with cryptogenic ischemic stroke or TIA. Medical therapy was categorized as antiplatelet therapy, anticoagulation, or mixed medical therapy according to the treatment information reported in the original studies. Integrating direct and indirect evidence, PFO closure ranked highest for reducing recurrent cerebrovascular ischemic events, followed by mixed medical therapy, anticoagulation, and antiplatelet therapy. For bleeding outcomes, PFO closure showed the lowest overall risk, whereas anticoagulation was associated with relatively higher bleeding risk. Model fit/consistency indices (DIC difference <5) support the robustness of these conclusions. This evidence map can help clinicians make individualized decisions within different frameworks of patient risk and preference.

Compared with previous meta-analyses, our study provides several additional contributions. Mir et al. performed a systematic review and network meta-analysis comparing PFO closure, antiplatelet therapy, and anticoagulation in patients aged <60 years with PFO and cryptogenic stroke (36). Saber et al. also conducted a network meta-analysis based on randomized evidence and suggested that PFO closure and oral anticoagulation were both associated with lower recurrent stroke risk than antiplatelet therapy, but with different safety profiles (37). Tsivgoulis et al. further evaluated percutaneous PFO closure for secondary stroke prevention and showed a reduction in recurrent ischemic events, accompanied by an increased risk of new-onset atrial fibrillation (37). In addition, Varotto et al. focused on device-level comparisons for preventing subsequent neurological events after PFO closure (38), and later examined the balance between recurrent stroke prevention and atrial fibrillation risk in a network meta-analysis (39). More recently, Hammad et al. updated the evidence comparing PFO closure with medical therapy for secondary prevention of stroke and TIA (40). Compared with these studies, the present analysis adds value in several specific ways. First, it incorporates both randomized controlled trials and observational cohorts, thereby expanding the evidence base while preserving the clinical focus on studies that included a PFO-closure comparison. Second, it excludes drug-only comparisons without a PFO closure arm, which helps keep the network aligned with the clinical decision between closure and non-closure strategies. Third, it categorizes medical therapy as antiplatelet therapy, anticoagulation, or mixed medical therapy only when these arms were reported within eligible PFO-closure comparative studies. Fourth, it evaluates recurrent cerebrovascular ischemic events and bleeding outcomes within the same four-node framework, allowing the efficacy and safety profiles to be considered together. Therefore, the main contribution of this study is not only an updated estimate of PFO closure efficacy, but also a more transparent comparative benefit–risk framework for PFO closure versus medical therapy. At the same time, comparisons among medical therapies should still be interpreted as secondary indirect evidence rather than definitive evidence of superiority.

From a mechanistic perspective, the favorable efficacy of PFO closure is consistent with its ability to anatomically eliminate the right-to-left shunt and thereby interrupt the potential pathway for paradoxical embolism from the venous to the arterial circulation (3, 41). Anticoagulation has biological plausibility in this setting because it may reduce venous thrombus formation; however, available ESUS/PFO subgroup data do not establish a definitive clinical advantage over antiplatelet therapy, and any potential benefit must be balanced against bleeding risk (42, 43). Antiplatelet therapy may remain appropriate for selected patients, particularly when bleeding risk, contraindications, or patient preference favor a safer antithrombotic profile. With respect to safety, we did not include new-onset atrial fibrillation in the quantitative synthesis because the available data were insufficient and inconsistently reported; however, previous evidence suggests that early post-closure atrial fibrillation may occur, while the long-term burden appears less certain (44). Therefore, these mechanistic considerations support the clinical plausibility of our findings, but the main contribution of the present study remains the updated comparative benefit–risk assessment across eligible PFO-closure studies.

Several limitations merit attention. First, some primary studies did not distinguish major from non-major bleeding, constraining the precision and clinical interpretability of the pooled safety analysis. Second, although our quality assessment accounted for confounding control in observational studies (propensity scores/multivariable adjustment), residual confounding cannot be entirely excluded. Third, several studies reported only an overall medical-treatment arm; these studies were therefore coded as mixed medical therapy rather than being artificially split into antiplatelet or anticoagulation nodes. Fourth, between-study differences in baseline characteristics (age, high-risk anatomy, comorbidities), follow-up duration, device generations, and antithrombotic dosing strategies may contribute to unexplained heterogeneity. In particular, evidence for elderly patients remains limited, and the available closure data mainly reflect percutaneous rather than surgical closure. In addition, most studies did not provide sufficiently detailed event data to distinguish individual antiplatelet or anticoagulant drugs, including warfarin versus direct oral anticoagulants; therefore, drug-level analyses of recurrent stroke/TIA or bleeding events were not feasible. We also considered Trial Sequential Analysis (TSA) to evaluate the risk of random errors and the certainty of cumulative evidence. However, TSA is primarily designed for pairwise meta-analysis and is not readily applicable to the present network meta-analysis, which combined randomized and observational evidence across four intervention nodes and relied on both direct and indirect comparisons. Therefore, TSA was not performed. Instead, we assessed model fit and consistency using DIC, evaluated study quality and risk of bias, provided raw event data for transparency, and interpreted medical-therapy-node comparisons cautiously as secondary indirect estimates. To strengthen certainty, future research should include large, long-term RCTs with stratified validation in older adults and patients without high-risk anatomical features; in terms of safety, standardizing bleeding definitions and reporting, and harmonizing arrhythmia monitoring strategies will better delineate the safety spectrum. Methodologically, subsequent network meta-analyses should systematically test the transitivity/consistency assumptions and enhance stratified analyses and network meta-regression for key effect modifiers.

## 5 Conclusion

Overall, integrating the most up-to-date evidence, this study indicates that for appropriately selected patients with cryptogenic stroke/TIA and concomitant PFO, closure provides the strongest evidence and highest ranking for preventing recurrence, with an overall lower bleeding risk. When closure is not feasible or when patients and clinicians prefer a non-interventional strategy, the choice of antithrombotic therapy should be individualized according to ischemic recurrence risk, bleeding risk, patient preference, and clinical contraindications. The medical-therapy-node comparisons should be interpreted as secondary indirect estimates rather than definitive evidence of superiority among medical therapies alone. These findings provide clear evidence to support optimization of clinical pathways and guideline updates, and offer a more interpretable quantitative basis for patient-centered decision-making.

## Data Availability

The original contributions presented in the study are included in the article/Supplementary Material, further inquiries can be directed to the corresponding authors.
