# Supplementary material for: Patent foramen ovale closure versus medical therapy for prevention of recurrent ischemic events in patients with cryptogenic stroke or transient ischemic attack and documented patent foramen ovale: a systematic review and network meta-analysis of randomized controlled trials and observational studies
Source: Front Cardiovasc Med. 2026 Aug 12;13:1785684. doi: 10.3389/fcvm.2026.1785684 (PMC13507727; doi:10.3389/fcvm.2026.1785684)
Supplement: Supplementary file 1 [file Table1.docx]

**Supplementary Materials 1**-June 22, 2026

Search strategy

**Pubmed**

| Search number | Query | Results |
| --- | --- | --- |
| 4 | ((("Foramen Ovale, Patent"[Mesh]) OR (((Foramen Ovale, Patent[Title/Abstract]) OR (patent foramen ovale[Title/Abstract])) OR (PFO[Title/Abstract]))) AND (((("Stroke"[Mesh]) OR ("Brain Ischemia"[Mesh])) OR ("Ischemic Attack, Transient"[Mesh])) OR ((((((Stroke[Title/Abstract]) OR (Brain Ischemia[Title/Abstract])) OR (Ischemic Attack, Transient[Title/Abstract])) OR (cryptogenic stroke[Title/Abstract])) OR (cerebrovascular accident[Title/Abstract])) OR (TIA[Title/Abstract])))) AND (((secondary prevention[Title/Abstract]) OR (prophylaxis[Title/Abstract])) OR (recurrence prevention[Title/Abstract])) | 281 |
| 3 | ((secondary prevention[Title/Abstract]) OR (prophylaxis[Title/Abstract])) OR (recurrence prevention[Title/Abstract]) | 158,077 |
| 2 | ((("Stroke"[Mesh]) OR ("Brain Ischemia"[Mesh])) OR ("Ischemic Attack, Transient"[Mesh])) OR ((((((Stroke[Title/Abstract]) OR (Brain Ischemia[Title/Abstract])) OR (Ischemic Attack, Transient[Title/Abstract])) OR (cryptogenic stroke[Title/Abstract])) OR (cerebrovascular accident[Title/Abstract])) OR (TIA[Title/Abstract])) | 476,767 |
| 1 | ("Foramen Ovale, Patent"[Mesh]) OR (((Foramen Ovale, Patent[Title/Abstract]) OR (patent foramen ovale[Title/Abstract])) OR (PFO[Title/Abstract])) | 9,249 |

**Embase**

| No. | Query | Results |
| --- | --- | --- |
| #10 | #3 AND #8 AND #9 | 603 |
| #9 | 'secondary prevention':ti,ab OR prophylaxis:ti,ab OR 'recurrence prevention':ti,ab | 266062 |
| #8 | #4 OR #5 OR #6 OR #7 | 880836 |
| #7 | 'stroke':ti,ab OR 'brain ischemia':ti,ab OR 'transient ischemic attack':ti,ab OR 'cryptogenic stroke':ti,ab OR 'cerebrovascular accident':ti,ab OR tia:ti,ab | 637528 |
| #6 | 'transient ischemic attack'/exp | 58794 |
| #5 | 'brain ischemia'/exp | 243454 |
| #4 | 'stroke'/exp | 565935 |
| #3 | #1 OR #2 | 19068 |
| #2 | 'patent foramen ovale':ab,ti OR pfo:ab,ti | 14227 |
| #1 | 'patent foramen ovale'/exp | 13875 |

**Cochrane**

| ID | Search | Hits |
| --- | --- | --- |
| #1 | MeSH descriptor: [Foramen Ovale, Patent] explode all trees | 154 |
| #2 | (Foramen Ovale, Patent):ti,ab,kw OR (patent foramen ovale):ti,ab,kw OR (PFO):ti,ab,kw | 510 |
| #3 | #1 OR #2 | 510 |
| #4 | MeSH descriptor: [Stroke] explode all trees | 19633 |
| #5 | MeSH descriptor: [Brain Ischemia] explode all trees | 5958 |
| #6 | MeSH descriptor: [Ischemic Attack, Transient] explode all trees | 1190 |
| #7 | (Stroke):ti,ab,kw OR (Brain Ischemia):ti,ab,kw OR (Ischemic Attack, Transient):ti,ab,kw OR (cryptogenic stroke):ti,ab,kw OR (cerebrovascular accident):ti,ab,kw | 88146 |
| #8 | (TIA):ti,ab,kw | 2522 |
| #9 | #4 OR #5 OR #6 OR #7 OR #8 | 88828 |
| #10 | (secondary prevention):ti,ab,kw OR (prophylaxis):ti,ab,kw OR (recurrence prevention):ti,ab,kw | 87750 |
| #11 | #3 AND #9 AND #10 | 112 |

**Wos**

| # | Search Query | Results |
| --- | --- | --- |
| 1 | TS=("stroke" OR "cryptogenic stroke" OR "cerebrovascular accident" OR TIA) | 547953 |
| 2 | TS=("patent foramen ovale" OR PFO) | 12936 |
| 3 | TS=("secondary prevention" OR prophylaxis OR "recurrence prevention") | 182441 |
| 4 | #3 AND #2 AND #1 | 447 |
