# Supplementary material for: Patent foramen ovale closure versus medical therapy for prevention of recurrent ischemic events in patients with cryptogenic stroke or transient ischemic attack and documented patent foramen ovale: a systematic review and network meta-analysis of randomized controlled trials and observational studies
Source: Front Cardiovasc Med. 2026 Aug 12;13:1785684. doi: 10.3389/fcvm.2026.1785684 (PMC13507727; doi:10.3389/fcvm.2026.1785684)
Supplement: Supplementary file 2 [file Table2.docx]

**Supplementary Material 2. Raw event data for recurrent ischemic and bleeding outcomes**

| **Study** | **Treatment** | **Recurrent ischemic events, n/N** | **Bleeding events, n/N** |
| --- | --- | --- | --- |
| Mas JL et al., 2017 | PFO closure | 0/238 | 2/238 |
| Mas JL et al., 2017 | Antiplatelet therapy | 14/235 | 5/235 |
| Mas JL et al., 2017 | Anticoagulation | 3/187 | 10/187 |
| Lee PH et al., 2018 | PFO closure | 0/60 | 0/60 |
| Lee PH et al., 2018 | Mixed medical therapy | 6/60 | 2/60 |
| Furlan AJ et al., 2012 | PFO closure | 22/447 | 10/447 |
| Furlan AJ et al., 2012 | Mixed medical therapy | 29/462 | 4/462 |
| Saver JL et al., 2017 | PFO closure | 18/499 | 2/499 |
| Saver JL et al., 2017 | Mixed medical therapy | 28/481 | 0/481 |
| Sondergaard L et al., 2017 | PFO closure | 6/441 | 8/441 |
| Sondergaard L et al., 2017 | Antiplatelet therapy | 12/223 | 6/223 |
| Meier B et al., 2013 | PFO closure | 7/204 | 8/204 |
| Meier B et al., 2013 | Mixed medical therapy | 11/210 | 12/210 |
| Casaubon L et al., 2007 | PFO closure | 4/60 | Not included in safety analysis |
| Casaubon L et al., 2007 | Antiplatelet therapy | 9/41 | Not included in safety analysis |
| Casaubon L et al., 2007 | Anticoagulation | 3/20 | Not included in safety analysis |
| Paciaroni M et al., 2011 | PFO closure | 7/121 | Not included in safety analysis |
| Paciaroni M et al., 2011 | Antiplatelet therapy | 6/93 | Not included in safety analysis |
| Paciaroni M et al., 2011 | Anticoagulation | 4/24 | Not included in safety analysis |
| Schuchlenz HW et al., 2005 | PFO closure | 2/167 | 4/167 |
| Schuchlenz HW et al., 2005 | Antiplatelet therapy | 24/66 | 0/66 |
| Schuchlenz HW et al., 2005 | Anticoagulation | 7/47 | 7/47 |
| Thanopoulos BV et al., 2006 | PFO closure | 0/48 | 0/48 |
| Thanopoulos BV et al., 2006 | Antiplatelet therapy | 6/44 | 9/44 |
| Windecker S et al., 2004 | PFO closure | 9/150 | Not included in safety analysis |
| Windecker S et al., 2004 | Antiplatelet therapy | 15/79 | Not included in safety analysis |
| Windecker S et al., 2004 | Anticoagulation | 6/79 | Not included in safety analysis |
| Low CE et al., 2023 | PFO closure | 2/84 | Not included in safety analysis |
| Low CE et al., 2023 | Mixed medical therapy | 7/148 | Not included in safety analysis |
| Lee PH et al., 2024 | PFO closure | 15/161 | 6/161 |
| Lee PH et al., 2024 | Mixed medical therapy | 51/276 | 18/276 |
| Chen PL et al., 2023 | PFO closure | 6/97 | Not included in safety analysis |
| Chen PL et al., 2023 | Mixed medical therapy | 13/76 | Not included in safety analysis |
| Pezzini A et al., 2016 | PFO closure | 15/206 | Not included in safety analysis |
| Pezzini A et al., 2016 | Mixed medical therapy | 33/315 | Not included in safety analysis |
| Danese A et al., 2017 | PFO closure | 0/77 | Not included in safety analysis |
| Danese A et al., 2017 | Mixed medical therapy | 2/82 | Not included in safety analysis |
| Liu K et al., 2020 | PFO closure | 15/383 | Not included in safety analysis |
| Liu K et al., 2020 | Mixed medical therapy | 44/208 | Not included in safety analysis |
| Poli S et al., 2021 | PFO closure | 6/146 | 0/146 |
| Poli S et al., 2021 | Mixed medical therapy | 1/90 | 0/90 |

Abbreviations: NMA, network meta-analysis; PFO, patent foramen ovale.
