# Supplementary material for: Patent foramen ovale closure versus medical therapy for prevention of recurrent ischemic events in patients with cryptogenic stroke or transient ischemic attack and documented patent foramen ovale: a systematic review and network meta-analysis of randomized controlled trials and observational studies
Source: Front Cardiovasc Med. 2026 Aug 12;13:1785684. doi: 10.3389/fcvm.2026.1785684 (PMC13507727; doi:10.3389/fcvm.2026.1785684)
Supplement: Supplementary file 3 [file Table3.docx]

**Supplementary Material 3.** Model-fit assessment using deviance information criterion (DIC)

| Outcome | Consistency DIC | Inconsistency DIC | Difference in DIC |
| --- | --- | --- | --- |
| Primary efficacy outcome | 19.83 | 19.84 | 0.01 |
| Safety outcome: bleeding events | 9.36 | 9.53 | 0.18 |
